# Transgenic expression of delta-6 and delta-15 fatty acid desaturases enhances omega-3 polyunsaturated fatty acid accumulation in *Synechocystis* sp. PCC6803

**DOI:** 10.1186/1754-6834-7-32

**Published:** 2014-03-01

**Authors:** Gao Chen, Shujie Qu, Qiang Wang, Fei Bian, Zhenying Peng, Yan Zhang, Haitao Ge, Jinhui Yu, Ning Xuan, Yuping Bi, Qingfang He

**Affiliations:** 1 Biotechnology Research Center, Shandong Academy of Agricultural Sciences, Jinan 250100, P. R. China; 2 Shandong Provincial Key Laboratory of Genetic Improvement, Ecology and Physiology of Crops, Jinan 250100, P. R. China; 3 Department of Applied Science, University of Arkansas, Little Rock, Arkansas 72204, USA; 4 Key Laboratory of Algal Biology, Institute of Hydrobiology, Chinese Academy of Sciences, Wuhan 430072, P. R. China; 5 Test Base Service Center, Shandong Academy of Agricultural Sciences, Jinan 250100, P. R. China; 6 School of Life Science, Shandong University, Jinan 250100, P. R. China

**Keywords:** Omega-3 polyunsaturated fatty acids, Gene expression, Fatty acid desaturase, *Synechocystis* sp. PCC6803

## Abstract

**Background:**

Polyunsaturated fatty acids (PUFAs), which contain two or more double bonds in their backbone, are the focus of intensive global research, because of their nutritional value, medicinal applications, and potential use as biofuel. However, the ability to produce these economically important compounds is limited, because it is both expensive and technically challenging to separate omega-3 polyunsaturated fatty acids (ω-3 PUFAs) from natural oils. Although the biosynthetic pathways of some plant and microalgal ω-3 PUFAs have been deciphered, current understanding of the correlation between fatty acid desaturase content and fatty acid synthesis in *Synechocystis* sp. PCC6803 is incomplete.

**Results:**

We constructed a series of homologous vectors for the endogenous and exogenous expression of Δ6 and Δ15 fatty acid desaturases under the control of the photosynthesis *psbA2* promoter in transgenic *Synechocystis* sp. PCC6803. We generated six homologous recombinants, harboring various fatty acid desaturase genes from *Synechocystis* sp. PCC6803, *Gibberella fujikuroi* and *Mortierella alpina.* These lines produced up to 8.9 mg/l of α-linolenic acid (ALA) and 4.1 mg/l of stearidonic acid (SDA), which are more than six times the corresponding wild-type levels, at 20°C and 30°C. Thus, transgenic expression of Δ6 and Δ15 fatty acid desaturases enhances the accumulation of specific ω-3 PUFAs in *Synechocystis* sp. PCC6803.

**Conclusions:**

In the blue-green alga *Synechocystis* sp. PCC6803, overexpression of endogenous and exogenous genes encoding PUFA desaturases markedly increased accumulation of ALA and SDA and decreased accumulation of linoleic acid and γ-linolenic acid. This study lays the foundation for increasing the fatty acid content of cyanobacteria and, ultimately, for producing nutritional and medicinal products with high levels of essential ω-3 PUFAs.

## Background

Polyunsaturated fatty acids (PUFAs) are hydrocarbon chains that are 18 to 22 carbons in length, and have two or more double bonds in their backbone structure. PUFAs are classified as omega-3 (ω-3) and omega-6 (ω-6) fatty acids (FAs), based on the position of the first double bond from the methyl end. PUFAs, especially ω-3 PUFAs, are essential dietary molecules with potential medicinal applications, and have recently become the focus of intensive research. The importance of ω-3 PUFAs in disease prevention and human nutrition has been scientifically recognized for a number of years. Intake of ω-3 PUFAs is reported to reduce the risk of cardiovascular disease [1-4], neurological disorder [5], inflammation [6-8], and cancer [9]. Stearidonic acid (SDA, 18:4n-3) is a highly unsaturated plant-based ω-3 PUFAs with potential health benefits. SDA is a metabolic intermediate in the conversion of α-linolenic acid (ALA, 18:3n-3) to eicosapentaenoic acid (EPA, 20:5n-3) and docosahexaenioc acid (DHA, 22:6n-3), and is more stable than either EPA or DHA because of its lower unsaturation index. Because SDA is readily converted into EPA and DHA upon consumption, it may potentially be used to increase blood levels of ω-3 PUFAs [10,11]. Indeed, ingestion of vegetable oils enriched in SDA has been shown to increase EPA concentrations in tissue [12], and Kawabata *et al.*[13] demonstrated that increasing the consumption of SDA-containing soybean oil modifies the lipid and FA profiles in body fats. Becasue ω-6 PUFAs are not readily converted into ω-3 PUFAs, the ratio of ω-6 to ω-3 PUFAs is largely determined by dietary intake of FAs [14]. Modern diets tend to contain excessive levels of ω-6 FAs, such as linoleic acid (LA) and γ-linolenic acid (GLA), and low levels of ω-3 FAs, such as ALA and SDA [15]. Ancestral dietary fat compositions exhibited a ω-6 to ω-3 ratio of 2:1 to 4:1, but this ratio can be as high as 10:1 in modern diets [16]. These imbalances can increase the risk of hypertension [17], cardiovascular disease [18], rheumatoid arthritis [19-21], and inflammatory and autoimmune disease [22,23].

PUFAs also have potential applications in biofuel production. Genetic and metabolic engineering techniques can be used to increase targeted lipid content (specific or total lipid), and reduce the cost of microalgal diesel production [24-26]. The FA composition of the oil determines the storage stability of biodiesel [27]. Although PUFAs are more susceptible to oxidation, a higher percentage of unsaturated FAs in the feedstock oil results in biodiesel with improved cold-flow properties [28]. Therefore, research that aims to improve production of PUFAs will have a positive impact on food security, human nutrition, and biodiesel production.

Traditionally, ω-3 PUFA products have been derived mainly from fish oil and shellfish [29,30]. However, overfishing has seriously depleted resources [31], and it is expensive and technically challenging to extract ω-3 PUFAs from their natural sources. There is thus an urgent need to identify alternative and sustainable sources of ω-3 PUFAs [32-34]. Oils are mainly composed of palmitic acid (PA, C16:0), oleic acid (OA, C18:1n-9), ALA, and SDA [35,36], and enhancing the production of these individual FAs would improve oil production.

Metabolic engineering provides a powerful and effective approach for enhancing production of PUFAs. The FA biosynthetic pathways in higher plants and microalgae have been well studied, and much research has recently focused on developing metabolic engineering methods to increase the FA content of microalgae [37-41] and oilseed crops [42,43]. Several groups have generated transgenic plants that synthesize and accumulate PUFAs in storage seed oils [44-48]. Reddy and Thomas used the constitutive Cauliflower Mosaic Virus (CaMV) 35S promoter to control the expression of a Δ6 desaturase (isolated from the cyanobacterium *Synechocystis*) in *Nicotiana tabacum* (tobacco plant), which resulted in accumulation of low levels of GLA and SDA in the transgenic leaves [49]. Much higher levels of GLA and SDA accumulation (accounting for 20% of total FAs) were obtained by expressing the Δ6 desaturase from *Borago officinalis* in tobacco [50] and oilseed crops [51]. Meanwhile, the biotechnology companies Monsanto and Solae LLC successfully bred transgenic soybean plants producing 5 to 8% GLA and 15 to 30% SDA. These transgenic soybean plants have now been commercialized after passing a safety assessment [52]. Ruiz-López *et al.*[53] expressed a Δ6 desaturase from *Primula vialii* in linseed, and the transgenic plants accumulated 13.4% SDA without any GLA in their lipids. More recently, transgenic plants producing EPA and DHA, generated by various approaches, have been reported by several groups; however, the levels of PUFAs achieved are not nearly as high as those in fish oil. Other attempts to increase PUFA content in transgenic plants include optimization of various aspects, such as identification of improved desaturases or of the specific acyl-exchange mechanisms of PUFAs, maintenance of a continuous flux of substrates through the PUFA biosynthesis pathways without significant negative influence on triacylglycerol (TAG), and co-expression of transgenes [14,54].

Cyanobacteria are prokaryotes that are able to produce valuable metabolites using energy from sunlight. The blue-green alga *Synechocystis* sp. PCC6803, a facultative phototroph, is a unicellular cyanobacterium that is ideal for studying FA accumulation, and it has been explored as a vector for commercial manufacture of biofuels and oils [55]. In this study, we cloned the *Δ6* and *Δ15* FA desaturases of *Synechocystis* sp. PCC6803, optimized the codon usage of the *M. alpina Δ6* FA desaturase and the *G. fujikuroi* bifunctional *Δ12/Δ15* FA desaturase for optimal translation in *Synechocystis* sp. PCC6803, and expressed these genes in *Synechocystis* sp. PCC6803. We then analyzed the FA content and composition by gas chromatography (GC), and determined the correlations between the variety of FA desaturase and FA synthesis. We also investigated the expression patterns of endogenous and exogenous Δ6 and Δ15 FA desaturases at 20°C and 30°C, respectively, using quantitative real-time PCR and immunoblot analysis. By overexpression of endogenous and exogenous genes encoding PUFA desaturases, we markedly increased the accumulation of ALA and SDA and decreased the accumulation of LA and GLA in this cyanobacterium.

## Results and discussion

### Transgenic expression of *Δ6 and Δ15* genes in *Synechocystis*

**Figure 1:**
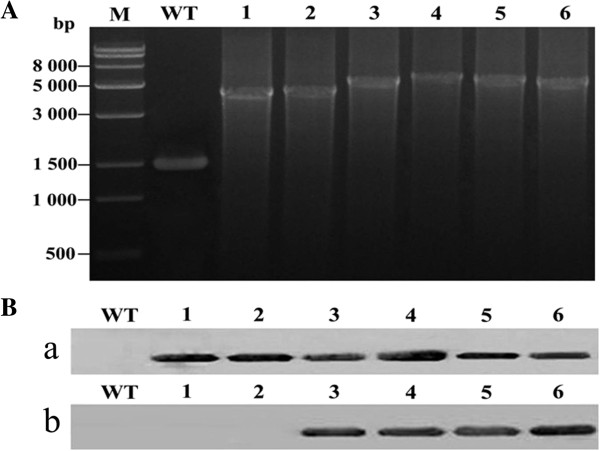
**PCR and immunoblot analysis of wild-type (WT) *Synechocystis *and desaturase transformants. (A)** PCR analysis of transgenic *Synechocystis*, in which a fragment of *psbA2* was deleted and replaced with various exogenous genes. M, Trans15k DNA marker; WT, wild-type *Synechocystis* sp. PCC6803; lane 1, pSDSy15; lane 2, pSDGf1215; lane 3, pSDSy15Sy6; lane 4, pSDGf1215Ma6; lane 5, pSDSy15Ma6; lane 6, pSDGf1215Sy6. The primers used in the PCR analysis (psbA2 promoter-F and psbA2-R) are described in the Methods section, and were combined to amplify the *psbA2* fragments (1.5 kb). **(B)** Immunoblot analysis of WT *Synechocystis* and desaturase transformants using **(a)** Flag tag and **(b)** His tag antibodies. Lane 1, WT *Synechocystis* sp. PCC6803; lanes 2–7, pSDSy15, pSDGf1215, pSDSy15Sy6, pSDGf1215Ma6, pSDSy15Ma6, and pSDGf1215Sy6 *Synechocystis* transformants, respectively.

Transformants were selected by subculture on BG-11 solid medium containing kanamycin 50 μg/ml. The complete segregation of the transformants was confirmed by PCR (Figure 1A). Amplification of the *Δ15* or *Δ6* gene fragment with the *psbA2* promoter, T1T2 terminator, and kanamycin cassette, coupled with the lack of amplification of the DNA fragment (1.5 kb) of the wild-type (WT) *psbA2* gene and promoter, indicated that the *Synechocystis* lines were indeed the expected transformants.

To detect the expression of *Δ15* or *Δ6* in transformants, we exposed the WT and *Δ6*/*Δ15* transformant cells to temperatures of 30°C. Immunoblot analysis confirmed the expression of the Δ6 and Δ15 desaturases, cloned in-frame with the *psbA2* promoter and T1T2 terminator, in the *Synechocystis* transformants. Immunoblot analysis of the Δ6 and Δ15 desaturases using His tag and Flag tag antibodies, respectively, confirmed the presence of the Δ6 and Δ15 proteins in *Synechocysits* transformants (Figure 1B). The Δ6 and Δ15 proteins localized to the soluble fraction of *Synechocystis* cell extracts.

### ALA and SDA production by transgenic cyanobacterial cells

The physicochemical properties of the cell membrane are reported to change rapidly with changes in temperature [44-47]. To study the effect of temperature on FA content, WT and mutant *Synechocystis* sp. PCC6803 cells were separately grown under mixotrophic conditions at 20 and 30°C and then the FA content in membranes was analyzed by GC (Table 1).

**Table 1:** **Fatty acid content of wild-type and transgenic *Synechocystis *** **sp. PCC6803**^**a–d**^

**Strain**	**T, °C**	**TFA, mg/g**	**FA content, %**					
			**C18:1**	**C18:2**	**C18:3n-6**	**C18:3n-3**	**C18:4**	**Others**
Wild type	30	75.201	7.07 ± 0.9	13.59 ± 1.0	16.26 ± 1.2	1.45 ± 0.2	1.20 ± 0.3	60.44 ± 4.3
	20	60.640	3.94 ± 0.2	16.75 ± 0.6	14.72 ± 1.3	2.53 ± 0.3	1.54 ± 0.2	60.53 ± 3.6
pSDSy15	30	50.755	2.73 ± 0.3	2.80 ± 0.5	0.30 ± 0.1	17.52 ± 2.3	9.11 ± 1.3	67.54 ± 7.3
	20	60.760	3.27 ± 0.7	1.94 ± 0.3	0.23 ± 0.1	23.05 ± 2.3	10.77 ± 1.6	60.73 ± 5.7
pSDGf1215	30	67.085	3.17 ± 0.3	15.56 ± 1.3	13.79 ± 2.0	1.78 ± 0.3	1.20 ± 0.2	64.50 ± 5.5
	20	66.990	5.73 ± 1.3	17.67 ± 1.0	12.71 ± 0.8	1.63 ± 0.1	0.96 ± 0.2	61.29 ± 8.3
pSDSy15Sy6	30	63.071	4.10 ± 0.8	2.57 ± 0.6	0.21 ± 0.1	23.64 ± 3.4	7.76 ± 0.7	61.72 ± 8.1
	20	57.130	2.28 ± 0.2	1.30 ± 0.3	0.19 ± 0.1	16.35 ± 1.9	13.12 ± 1.3	66.77 ± 6.5
pSDGf1215M6	30	68.803	3.11 ± 0.4	17.21 ± 2.3	14.45 ± 1.6	2.05 ± 0.2	1.18 ± 0.1	61.98 ± 5.3
	20	75.657	1.20 ± 0.1	10.65 ± 2.3	17.16 ± 1.6	3.02 ± 0.5	1.70 ± 0.1	66.27 ± 5.9
pSDSy15Ma6	30	61.480	3.45 ± 0.2	0.70 ± 0.1	0.21 ± 0.1	17.79 ± 2.3	11.10 ± 1.5	66.76 ± 3.9
	20	35.190	2.03 ± 0.2	1.22 ± 0.3	0.14 ± 0.1	14.83 ± 2.9	12.36 ± 1.5	69.42 ± 5.3
pSDGf1215Sy6	30	59.192	2.80 ± 0.2	15.98 ± 0.6	13.95 ± 0.7	2.40 ± 0.4	1.57 ± 0.1	63.32 ± 6.0
	20	53.651	6.75 ± 1.2	17.17 ± 2.0	10.81 ± 0.9	2.11 ± 0.6	0.89 ± 0.1	62.27 ± 8.7

FA, fatty acid; T, Temperature; TFA, Total fatty acid.

^a^Values are means of triplicate experiments.

^b^Cells were grown under a light intensity of 40 μmol photon/m^2^/s for 10 d in BG-11 medium.

^c^The membrane lipids were extracted from wild-type and genetically engineered *Synechocystis* sp. PCC6803.

^d^The enzymes overexpressed are indicated in parentheses (Sy15: Δ15 FA desaturase from *Synechocystis* sp. PCC6803; Sy6: Δ6 FA desaturase from *Synechocystis* sp. PCC6803; Gf1215: bifunctional Δ12/Δ15 FA desaturase from *Gibberella fujikuroi*; Ma6: Δ6 FA desaturase from *Mortierella alpina*).

In WT cells grown under mixotrophic conditions at 30°C, the total FA content was about 75.20 mg/g (dry weight). Compared with the WT, the FA content of transgenic *Synechocystis* sp. PCC6803 overexpressing *Synechocystis* Δ15 desaturase increased by 32.5%. In WT cells grown under mixotrophic conditions at 20°C, the total FA content was about 60.64 mg/g, but overexpression of *Synechocystis* Δ15 desaturase (SDSy15 vector) resulted in a slight increase in total FA content (0.2%). Compared with WT (see Additional file 1: Figure S2A), the C18:1, C18:2, and C18:3n-6 content of the transgenic cells was markedly lower, and the C18:3n-3 and C18:4 content markedly higher. For example, under mixotrophic cultivation at 30°C, C18:3n-3 increased from 1.45% to 17.52% and C18:4 increased from 1.20% to 9.11% (see Additional file 1: Figure S2B), whereas at 20°C C18:3n3 increased from 2.23% to 23.05% and C18:4 increased from 1.54% to 10.77% (see Additional file 1: Figure S2A′, Figure S2B′). In our experiments, from 1000 ml of BG-11 medium under mixotrophic cultivation at 20°C, we obtained 0.63 g (dry weight) of *Synechocystis* cells overexpressing Δ15 desaturase. GC analysis showed that this line produced up to 8.9 mg/l and 4.1 mg/l of ALA and SDA, respectively. These results suggest that overexpression of *Synechocystis* Δ15 desaturase in *Synechocystis* sp. PCC6803 improves the yield of C18:3n-3 and C18:4, and decreases the yield of C18:2 and C18:3n-6. Furthermore, the total FA content of this transformant increased when the temperature was decreased from 30°C to 20°C.

Compared with the WT, the FA content also decreased in *Synechocystis* sp. PCC6803 lines in which *Synechocystis* Δ6 and Δ15 desaturases and (SDSy15Sy6 vector) or *M. alpina* Δ6 and *Synechocystis* Δ15 FA desaturase (SDSy15Ma6 vector) were tandemly expressed. The total FA content of lines transformed with SDSy15Sy6 and SDSy15Ma6 was about 63.07 mg/g and 61.48 mg/g, respectively, at 30°C, which decreased by 16.1% and 18.3% under mixotrophic cultivation, and 57.13 mg/g and 35.19 mg/g at 20°C, which decreased by 41.9% under mixotrophic cultivation. Compared with the WT, the C18:1, C18:2, and C18:3n-6 content decreased and the C18:3n-3 and C18:4 content markedly increased in pSDSy15Sy6 and pSDSy15Ma6. In lines transformed with SDSy15Sy6, C18:3n-3 increased from 1.45% to 23.64% and C18:4 increased from 1.20% to 7.76% (see Additional file 1: Figure S2C), while in lines transformed with SDSy15Ma6, C18:3n-3 increased from 1.45% to 17.79% and C18:4 increased from 1.20% to 11.10% (see Additional file 1: Figure S2D) under mixotrophic cultivation at 30°C relative to the WT control. By contrast, at 20°C in lines transformed with SDSy15Sy6, C18:3n-3 content increased from 2.23% to 16.35% and C18:4 increased from 1.54% to 13.12% (see Additional file 1: Figure S2C′), while in lines transformed with SDSy15Ma6 under mixotrophic cultivation at 20°C, C18:3n-3 increased from 2.23% to 12.36% and C18:4 increased from 1.54% to 14.83% (see Additional file 1: Figure S2D′).

These results show that tandem expression of Δ6 and Δ15 desaturases in *Synechocystis* sp. PCC6803 markedly increases the yield of C18:3n-3 and C18:4. Furthermore, C18:4 content increased as the temperature decreased, indicating that a reduction in temperature promotes the expression of Δ6 desaturase, which converts ALA to SDA. These results also suggest that production of PUFAs is greater in transgenic *Synechocystis* organisms cultivated at 20°C than in those cultivated at higher temperatures.

In addition, we generated three more types of transgenic *Synechocystis* sp. PCC6803, namely pSDGf1215, which overexpressed *G. fujikuroi* bifunctional Δ12/Δ15 FA desaturase; pSDGf1215Ma6, which tandemly expressed *M. alpina* Δ6 FA desaturase and *G. fujikuroi* bifunctional Δ12/Δ15 FA desaturase; and pSDGf1215Sy6, which tandemly expressed *Synechocystis* Δ6 FA desaturase and *G. fujikuroi* bifunctional Δ12/Δ15 FA desaturase. Under mixotrophic cultivation at 30°C, the total FA content of the lines transformed with SDGf1215, SDGf1215Ma6, and SDGf1215Sy6 was 67.09, 68.80, and 59.19 mg/g, respectively, which was 10.8%, 8.5%, and 21.3%, respectively, less than that of the WT. Under mixotrophic cultivation at 20°C, the total FA content of pSDGf1215 and pSDGf1215Ma6 was 66.99 and 75.66 mg/g, respectively, which was 10.5% and 24.8% greater than that of the WT. The total FA content of pSDGf1215Sy6 was 53.65 mg/g, which was 11.0% less than that of the WT. Whereas the C18:1 and C18:2 content was much higher in the WT than in the transgenic lines, the C18:3n-6, C18:3n-3, and C18:4 content was markedly lower in the WT than in the transgenic lines.

Overexpression of endogenous Δ6 and Δ15 desaturases in *Synechocystis* sp. PCC6803 greatly enhanced PUFA accumulation. By contrast, heterologous expression of *M. alpina* Δ6 FA desaturase and *G. fujikuroi* bifunctional Δ12/Δ15 FA desaturase in *Synechocystis* sp. PCC6803 had no obvious effect. Although both of these desaturases were previously shown to have obvious effects on FA accumulation in non-photosynthetic microbes and plants [48,56], we found that overexpression of these two exogenous enzymes had no significant effect on PUFA accumulation in *Synechocystis* sp. PCC6803.

### Effects of temperature on cell growth and FA composition of transgenic *Synechocystis*

**Figure 2:**
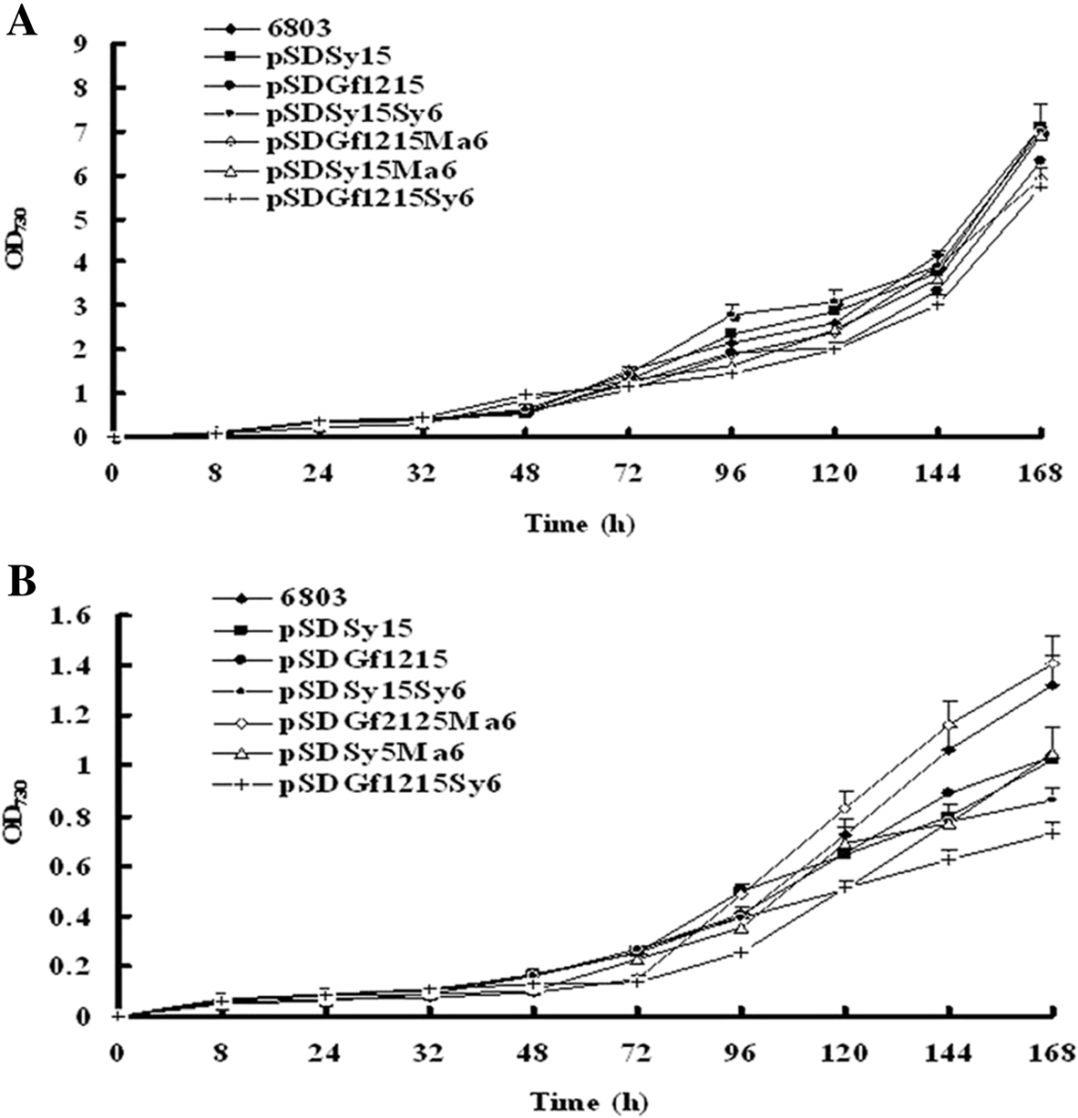
**Growth curves of wild-type and transgenic *Synechocystis.*** Cells were grown under mixotrophic conditions at **(A)** 30°C or **(B)** 20°C. Cultures were grown in BG-11 medium and bubbled with air under an illumination of 40 μmol photons/m^2^/s. The optical density of cells at 730 nm was measured at the indicated time points. Values are means ± SD (bars) of three independent experiments conducted on different days. Absence of a bar indicates that the SD falls within the symbol.

Temperature has a large effect on the types of FAs produced by microalgae, and the composition of FAs, in turn, affects the physiology of the organism by changing the rate of chemical reactions and the stability of cellular components [57,58]. Several studies suggest that the exponential growth rates of many microalgal species increase in response to elevated growth temperatures, up to an optimal temperature, and then decline once structural integrity has been lost [58,59]. To preserve the structural integrity of the cell, organisms regulate lipid composition to maintain proper membrane fluidity at different temperatures [58]. In response to cold stress, several microalgal species enhance the biosynthesis and accumulation of total FAs, which also results an increase in the content of PUFAs at low temperatures [47]. To test whether overexpression of the exogenous and endogenous desaturases affects the growth and FA content of transgenic *Synechocystis*, we examined photoautotrophic growth and FA variation of WT and transgenic *Synechocystis* cells at 30°C and 20°C in liquid medium (Figure 2).

The suspension cultures grown at 30°C were diluted to an OD_730 nm_ of 0.05, and were further incubated at 20°C and 30°C. We did not detect a significant change in the growth of WT and transgenic *Synechocystis* under mixotrophic cultivation at 30°C and 40 μmol photon/m^2^/s (Figure 2A), whereas growth of both WT and transgenic *Synechocystis* was much slower at 20°C than at 30°C (Figure 2B). The growth rates of pSDGf1215Ma6 and pSDGf1215Sy6 were markedly lower than those of the WT and other transgenic *Synechocystis* (pSDSy15, pSDGf1215, pSDSy15Sy6, and pSDSy15Ma6) when cells were grown at 20°C. It is unknown why overexpression of both Gf1215 and Ma6 or Sy6 affects cell growth; however, the resulting differences in lipid composition may affect the resistance of the cells to the cold. Although the transgenic *Synechocystis* strains had a higher ratio of C18:4 at 20°C, they grew much slower than at 30°C. Therefore, for optimal production of PUFAs, the transgenic *Synechocystis* should be cultivated at 30°C.

We found that reducing the temperature from 30°C to 20°C significantly altered FA composition in *Synechocystis* cells (Table 1). The reduction in temperature was accompanied by a decrease in the OA and GLA content, but an increase in LA, ALA, and SDA. Consistent with previous reports [60,61], these findings indicate that Δ6, Δ12, and Δ15 desaturases are more active at 20°C than at 30°C. The lower temperature did not affect the Δ9 desaturase activity, but the increase in the Δ12 desaturase activity caused an increase in LA at the expense of OA at 20°C. Activities of both the Δ6 and Δ15 desaturases increased at 20°C, which increased the content of ALA and SDA in both the WT and transgenic *Synechocystis* cells (pSDSy15, pSDSy15Sy6, and pSDSy15Ma6), and thereby enhanced production of ω-3 PUFAs. The reason why GLA level decreases at 20°C is somewhat complex, as the increase in the Δ6 desaturase activity and LA level would be expected to increase GLA level at 20°C. However, the decrease in GLA could be attributable, at least in part, to the competition between the Δ6 desaturases (responsible for GLA synthesis) and Δ15 desaturases (responsible for ALA synthesis) for their common substrate LA.

Biodiesel fuel derived from fats and oils has several advantages over petro-diesel. Although saturated fat is considered very suitable for the production of biodiesel, because of its oxidative stability and low levels of hydrocarbon and carbon monoxide emissions upon combustion [62], the biodiesel made from saturated fats tends to have a high melting point, and therefore, gelling of fuel may occur under cold weather, resulting in incomplete combustion and elevated mono-nitrogen oxide (NO_x_) emissions [63]. By contrast, biodiesel made from unsaturated fats has a lower gelling point. Therefore, unsaturated fats are an excellent source of biodiesel in cold climates [28]. With the addition of oxidative stabilizers, biodiesel made from highly unsaturated fats can be safely used as fuel.

## Conclusions

In this study, we successfully constructed six homologous recombination vectors harboring genes encoding polyunsaturated FA desaturases, and expressed these genes in the blue-green alga *Synechocystis* sp. PCC6803 to enhance the production of ALA and SDA. We analyzed the composition and content of FAs produced under different temperatures. These results expand the current understanding of the role of FA desaturases and the molecular mechanisms that underlie PUFA accumulation at different temperatures in *Synechocystis*. The transgenic *Synechocystis* lines generated in this study produced 8.9 mg/l and 4.1 mg/l of ALA and SDA, respectively, which was more than six-fold that produced by the WT. This is the first report to show that ALA and SDA production can be increased and LA and GLA production decreased using homologous recombinants expressing endogenous and exogenous Δ6 and Δ15 FA desaturases driven by the photosynthesis *psbA2* promoter. This study lays the foundation for increasing FA content in cyanobacteria and for producing large amounts of PUFAs using cyanobacteria.

## Methods

### Strains and growth conditions

The cyanobacterium *Synechocystis* sp. PCC6803 was obtained from the Freshwater Algae Culture Collection of the Institute of Hydrobiology, Chinese Academy of Sciences. *Synechocystis* sp. PCC6803 was cultivated in BG-11 medium (5 mM glucose) at 30°C [64]. For solid BG-11 medium, 1.5% (w/v) Difco Bacto-agar (Becton Dickinson, Sparks, MD, USA), 0.3% (w/v) sodium thiosulfate, and 10 mM TES (2-[(2-hydroxy-1, 1-bis (hydroxymethyl) ethyl) amino] ethanesulfonic acid pH 8.2) were added. The culture was bubbled with air under a light intensity of 40 μmol photon/m^2^/s. Transformed strains were selected by addition of 50 μg/ml kanamycin (Dingguo Company, Beijing, China) to the liquid and solid BG-11 media.

### Genomic DNA extraction

To extract genomic DNA from *Synechocystis* sp. PCC6803, 100 ml of *Synechocystis* culture (OD_730nm_ = 1.5) was extracted with phenol:chloroform (1:1, v/v) [65], and RNA was then removed using RNase A (TransGen Biotech, Beijing, China).

### Cloning of *Δ6 and Δ15* genes

The *Δ15* and *Δ16* genes of *Synechocystis* sp. PCC6803 were amplified by PCR. Genomic DNA was used as template and primers Delta 6 and Delta 15 (Table 2) were used to amplify *Δ6* and *Δ15*, respectively. The amplified fragments of *Δ6* (Sy6, 1.1 kb) and *Δ15* (Sy15, 1.1 kb) were cloned into a pGEM-T Easy Cloning Vector (Promega, Madison, WI) and sequenced at the Biotechnology Research Center, Shandong Academy of Agricultural Sciences (Jinan, China).

**Table 2:** **Primers used for PCR**^**a**^

**Primer**	**Sequence 5′ → 3′**
Delta 6-F	TAAGGAATTATAACCAAATGCTAACAGCGGAAAG
Delta 6-R	GTCCTGCAGTCAATGATGATGATGATGATGCGATGCTTTGCCCATGGCCT
Delta 15-F	TAAGGAATTATAACCAAATGCGTCTAGAAATTTCATCG
Delta 15-R	CGGCTGCAGTTACTTATCGTCGTCATCCTTGTAATCAGGTTTCTTTTGATATC
psbA2 promoter-F	GAT **GTCGAC** GCTTTAGCGTTCCAGTG
psbA2 promoter-R	CATTTGGTTATAAT TCCTTATGTAT
psbA2-F	CTT **CATATGCCGCGG** ATGACAACGACTCTCCAAC
psbA2-R	AGT **GAGCTC** TTAACCGTTGACAGCAGG

^a^Text that is bold and underlined indicates restriction enzyme sites (see text for details).

### Optimizing *Δ6* and *Δ15* desaturases and gene synthesis

The nucleotide sequences of *M. alpina Δ6* FA desaturase (Ma6, GenBank: AF110510) and *G. fujikuro*i bifunctional *Δ12/Δ15* FA desaturase (Gf1215, GenBank: DQ272516) were optimized for expression in *Synechocystis* using the codon usage database (DNA 2.0, Menlo Park, CA, USA) and synthesized (Sangon Co., Shanghai, China).

### Generation of fatty acid desaturase homologous recombination plasmids containing *Δ6* and *Δ15* desaturase genes

For the overexpression and heterologous expression of *Δ6* and *Δ15* genes (*desD* and *desB*, respectively) in *Synechocystis* sp. PCC6803, plasmid constructs were generated in which a His tag was added at the 3′ end of *Δ6* and a Flag tag at the 3′ end of *Δ15. Synechocystis psbA2* was replaced with His-tagged *Synechocystis Δ6* and *M. alpine Δ6,* Flag-tagged *Synechocystis Δ15*, and *G. fujikuroi Δ12Δ15* via double homologous recombination. The *psbA2* gene belongs to the *psbA* multi-gene family, and encodes a D1 protein of photosystem II. Mutants with inactivated *psbA2* are indistinguishable from the WT [66,67]. The *psbA2* promoter and open reading frame (ORF) were positioned upstream and downstream, respectively, of the FA desaturase genes in our constructs, and integrated into the shuttle vector, and the genomic *psbA2* genes of the transgenic cyanobacteria were inactivated by homologous recombination. Six homologous recombination plasmids were constructed to drive the production of SDA. To achieve this, the 500 bp fragment of *Synechocystis* genomic DNA upstream of the *psbA2* ORF was amplified by PCR using psbA2 promoter-F and psbA2 promoter-R primers (containing a *Sal*I site, underlined; Table 2). The 1.0 kb fragment of *Synechocystis* genomic DNA that encodes the *psbA2* ORF was amplified by PCR as the downstream region of the homologous recombination vector, using the primers psbA2-F (containing *Nde*I and *Sac*II sites, underlined; Table 2) and psbA2-R (containing the *Sac*I site, underlined; Table 2). The downstream fragment was cloned into the *Sac*II and *Sac*I sites of pBluescript SK plus T1T2, forming plasmid pST1T2. Then, the kanamycin resistance cassette carrying *npt* was cloned into the single *Bam*HI site of pST1T2, forming pSKT1T2. The upstream fragment was fused with Sy15, Gf1215, Sy6, or Ma6 by fusion PCR, and subsequently cloned into the *Sal*I site of pSKT1T2, to form plasmids SDSy15, SDGf1215, SDSy6, and SDMa6, respectivley. SDSy6 and SDMa6 were cloned into the *Nde*I and *Sac*II sites of SDSy15 and SDGf1215 to yield plasmids SDSy15Sy6, SDGf1215Ma6, SDSy15Ma6, and SDGf1215Sy6, respectivley. Six homologous recombination plasmid structures are listed (see Additional file 2: Figure S1).

### Transformation of *Synechocystis* sp. PCC6803

The *Synechocystis* sp. PCC6803 strain was transformed as described by He *et al.*[67] and Vermaas *et al*. [68]. *Synechocystis* sp. PCC6803 was grown in liquid BG-11 medium at 30°C until OD_730_ reached 0.6, then the cells were harvested by centrifugation and resuspended in fresh BG-11 to a density of OD_730_ = 4.8. Plasmid DNA was added to 500 μl of cell suspension and mixed gently, and the mixture was incubated at 30°C under low light for 6 h, and then spread on BG-11 agar plates. Transformants were selected by screening for resistance to 20 μg/ml kanamycin. Transformants were isolated after about 10 days of incubation, and subcultured on BG-11 agar plates containing 50 μg/ml kanamycin. The transformants were then grown in liquid culture for analysis.

### SDS-PAGE and immunoblot analysis

The crude extracts of WT and transformant cells were collected and dissolved in lysis buffer (1 ml of 40 mM Tris–HCl pH 8.0) with protease inhibitor (1 mM phenylmethanesulfonyl fluoride). After sonication, incubation, and centrifugation, the insoluble material was removed, and the supernatants were used for immunoblot analysis. The soluble proteins were separated on 12% SDS-PAGE gels and then blotted onto 0.45-μm PVDF membranes (Beijing CoWin Biotech Co., Ltd, Beijing, China), stained with antibody to His tag or Flag tag (1:5000, Beijing CoWin Biotech Co., Ltd., Beijing, China) for 1 h, and then treated with goat anti-rabbit IgG HRP at 1:5000 for 1 h. Cross-reactions between protein bands and antibodies were detected using an HRP-DAB Color Development Kit (Tiangen Biotech, Beijing, China), according to the manufacturer’s instructions.

### Lipid extraction and fatty acid methyl ester analysis of transgenic cyanobacteria

Membrane lipid extraction from WT and genetically engineered *Synechocystis* sp. PCC6803 was carried out as described by Bligh and Dyer [69]. The colonies were collected and transferred to 1000 ml autoclaved flasks, each containing 400 ml of sterile BG-11 medium, and grown for 10 days at a light intensity of 40 μmol photons/m^2^/s and a constant temperature of 30°C. Cultures were harvested when they reached OD_730_ = 3.0, then they were washed with distilled water and centrifuged (6000 × *g* for 10 minutes at room temperature) after washing to pellet cells; this washing and centrifugation was carried out three times in all. The wet cell samples were them incubated at 40°C to obtain 600 mg of dry cell paste, which was diluted with 4 ml chloroform/methanol (1:10 v/v), then a suspension of 1 ml hexane containing a C19:0 internal standard (1 mg/ml) was added. The mixture was heated at 80°C for 2 hours in a water bath, and after cooling, 5 ml of 7% potash was added and mixed. After 10 minues of incubation at room temperature, the mixture was centrifuged at 10,000 × *g* for 10 minutes. The supernatants (bacterial sample fatty acid methyl ester (FAME) eluate) were subjected to GC using the Elite-wax column in an ASXL instrument (Perkin-Elmer, Waltham, MA, USA). The flame-ionization detection temperature was 250°C, and the operating temperature was maintained at 220°C. The data are presented as the mean of three experiments for each sample.

## Abbreviations

ALA: α-Linolenic acid; DHA: Docosahexaenioc acid; EPA: Eicosapentaenoic acid; FA: Fatty acid; FAME: Fatty acid methyl ester; GC: Gas chromatography; GLA: γ-linolenic acid; LA: Linoleic acid; OA: Oleic acid; ORF: Open reading frame; PA: Palmitic acid; PUFAs: Polyunsaturated; PVDF: polyvinylidene difluoride; SDA: Stearidonic acid; TAG: triacylglycerol; WT: Wild-type.

## Competing interests

The authors declare that they have no competing interests.

## Authors’ contributions

GC was responsible for study conception and design, data collection and analysis, manuscript writing and final approval of the manuscript; QW for data collection and analysis, and final approval of the manuscript; SQ, FB, YZ, HG, JY, and NX for data collection and final approval of the manuscript; ZP for data analysis and final approval of the manuscript; YB for conception and data collection and final approval of the manuscript; and QH for conception and design, critical revision and manuscript writing, and final approval of the manuscript. All authors read and approved the final manuscript.

## Supplementary Material

Additional file 1: Figure S2 Gas chromatography analysis of fatty acids (FAs) in wild-type and transgenic *Synechocystis* under mixotrophic conditions. The C18 FA methyl esters are labeled. We extracted the lipid from wild-type and Δ6 and Δ15 transgenic *Synechocystis,* which were grown at **(A-D)** 30°C or **(A′-D′)** 20°C. Strains are: **(A and A′)** wild-type *Synechocystis* sp. PCC6803 **(B and B′)**; pSDSy15; **(C and C′)** pSDSy15Sy6 **(C and C′)**; and **(D and D′)** pSDSy15Ma6. Click here for file

Additional file 2: Figure S1 Structure of homologous recombination vectors harboring fatty acid (FA) desaturase genes. **(A)** SDSy15; overexpression of Δ15 FA desaturase from *Synechocystis* sp. PCC6803; **(B)** SDGf1215: overexpression of bifunctional Δ12/Δ15 FA desaturase from *Gibberella fujikuroi*; **(C)** SDSy15Sy6: overexpression of Δ6 and Δ15 FA desaturase from *Synechocystis* sp. PCC6803; **(D)** SDGf1215Ma6: overexpression of Δ6 FA desaturase from *Mortierella alpina* and of bifunctional Δ12/Δ15 FA desaturase from *G. fujikuroi*; **(E)** SDSy15Ma6: overexpression of Δ6 FA desaturase from *M. alpina* and of Δ15 FA desaturase from *Synechocystis* sp. PCC6803; and **(F)** SDGf1215Sy6: overexpression of Δ6 FA desaturase from *Synechocystis* sp. PCC6803 and of bifunctional Δ12/Δ15 FA desaturase from *G. fujikuroi*, Promoter, *psbA2* promoter; T1T2, *rrn*B (5S rRNA T1 and T2 transcription terminators from *Escherichia coli*); npt, neomycin phosphotransferase gene, conferring kanamycin resistance; pA2D, *psbA2* open reading frame (ORF) from *Synechocystis* sp. PCC6803. All Δ6 FA desaturases are tailed with a His tag and all Δ15 FA desaturases are tailed with a Flag tag. Click here for file
