# Supplementary figures and images for: Transgenic expression of delta-6 and delta-15 fatty acid desaturases enhances omega-3 polyunsaturated fatty acid accumulation in Synechocystis sp. PCC6803
Source: Biotechnol Biofuels. 2014 Mar 1;7:32. doi: 10.1186/1754-6834-7-32 (PMC3941260; doi:10.1186/1754-6834-7-32)

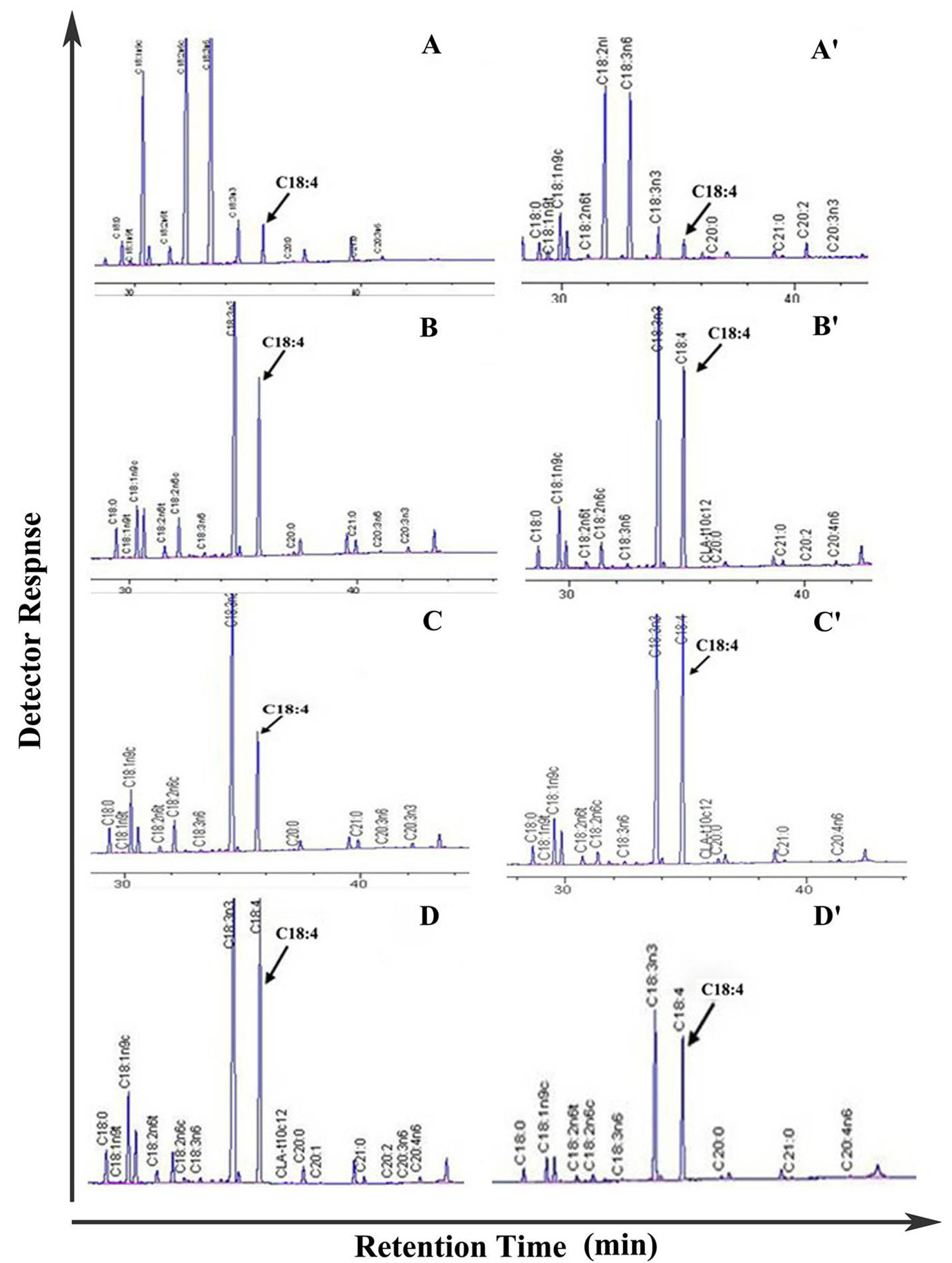

Supplement: Additional file 1: Figure S2 — Gas chromatography analysis of fatty acids (FAs) in wild-type and transgenic Synechocystis under mixotrophic conditions. The C18 FA methyl esters are labeled. We extracted the lipid from wild-type and Δ6 and Δ15 transgenic Synechocystis, which were grown at (A-D) 30°C or (A′-D′) 20°C. Strains are: (A and A′) wild-type Synechocystis sp. PCC6803 (B and B′); pSDSy15; (C and C′) pSDSy15Sy6 (C and C′); and (D and D′) pSDSy15Ma6. [file 1754-6834-7-32-S1.jpeg]

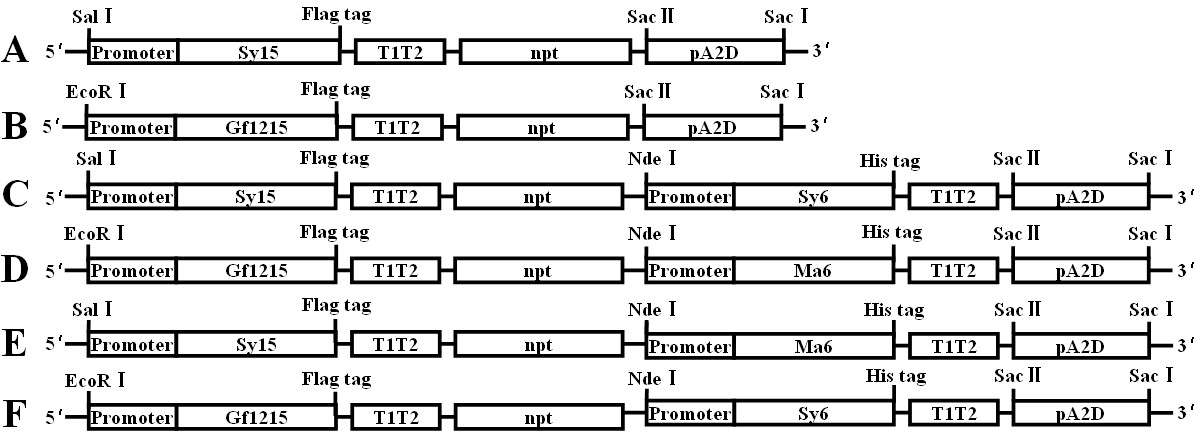

Supplement: Additional file 2: Figure S1 — Structure of homologous recombination vectors harboring fatty acid (FA) desaturase genes. (A) SDSy15; overexpression of Δ15 FA desaturase from Synechocystis sp. PCC6803; (B) SDGf1215: overexpression of bifunctional Δ12/Δ15 FA desaturase from Gibberella fujikuroi; (C) SDSy15Sy6: overexpression of Δ6 and Δ15 FA desaturase from Synechocystis sp. PCC6803; (D) SDGf1215Ma6: overexpression of Δ6 FA desaturase from Mortierella alpina and of bifunctional Δ12/Δ15 FA desaturase from G. fujikuroi; (E) SDSy15Ma6: overexpression of Δ6 FA desaturase from M. alpina and of Δ15 FA desaturase from Synechocystis sp. PCC6803; and (F) SDGf1215Sy6: overexpression of Δ6 FA desaturase from Synechocystis sp. PCC6803 and of bifunctional Δ12/Δ15 FA desaturase from G. fujikuroi, Promoter, psbA2 promoter; T1T2, rrnB (5S rRNA T1 and T2 transcription terminators from Escherichia coli); npt, neomycin phosphotransferase gene, conferring kanamycin resistance; pA2D, psbA2 open reading frame (ORF) from Synechocystis sp. PCC6803. All Δ6 FA desaturases are tailed with a His tag and all Δ15 FA desaturases are tailed with a Flag tag. [file 1754-6834-7-32-S2.jpeg]
